# Age-dependent changes of p53 and p63 immunoreactivities in the mouse hippocampus

**DOI:** 10.1186/s42826-019-0022-0

**Published:** 2019-10-29

**Authors:** Tae-Kyeong Lee, Young Eun Park, Cheol Woo Park, Bora Kim, Jae-Chul Lee, Joon Ha Park, Hyang-Ah Lee, Moo-Ho Won, Ji Hyeon Ahn

**Affiliations:** 1 Department of Neurobiology, School of Medicine, Kangwon National University, Chuncheon, Gangwon 24341 Republic of Korea; 2 Department of Anatomy, College of Korean Medicine, Dongguk University, Gyeongju, Gyeongbuk 38066 Republic of Korea; 3 Department of Obstetrics and Gynecology, School of Medicine, Kangwon National University, Chuncheon, Gangwon 24341 Republic of Korea; 4 Department of Biomedical Science, Research Institute of Bioscience and Biotechnology, Hallym University, Chuncheon, Gangwon 24252 Republic of Korea

**Keywords:** Aging process, Granule cells, Mouse, Hippocampus, p53, p63, Pyramidal neurons

## Abstract

P53 and its family member p63 play important roles in cellular senescence and organismal aging. In this study, p53 and p63 immunoreactivity were examined in the hippocampus of young, adult and aged mice by using immunohistochemistry. In addition, neuronal distribution and degeneration was examined by NeuN immunohistochemistry and fluoro-Jade B fluorescence staining. Strong p53 immunoreactivity was mainly expressed in pyramidal and granule cells of the hippocampus in young mice. p53 immunoreactivity in the pyramidal and granule cells was significantly reduced in the adult mice. In the aged mice, p53 immunoreactivity in the pyramidal and granule cells was more significantly decreased. p63 immunoreactivity was strong in the pyramidal and granule cells in the young mice. p63 immunoreactivity in these cells was apparently and gradually decreased with age, showing that p63 immunoreactivity in the aged granule cells was hardly shown. However, numbers of pyramidal neurons and granule cells were not significantly decreased in the aged mice with normal aging. Taken together, this study indicates that there are no degenerative neurons in the hippocampus during normal aging, showing that p53 and p63 immunoreactivity in hippocampal neurons was progressively reduced during normal aging, which might be closely related to the normal aging processes.

## Introduction

The p53 gene family is composed of a group of transcription factors including a key tumor suppressor p53, and its homologs p63 and p73 [1, 2]. It has widely been reported that the majority of human cancers show mutations that abrogate p53 network which is related to many anti-proliferative cellular responses in context-dependent manner [3–5]. In rodent and human brains, p53 and p63 expressions have been reported. p53 mRNA is expressed in the mouse [6] and rat [7] brain, and p63 mRNA and proteins are expressed in the human hippocampus and cortex [8]. Recently, researches on the activity of p53 or p63 in noncancer (normal) tissues in response to cerebral ischemia have been examined [9–11].

Aging is defined as progressive decline of normal body functions necessary for survival and reproduction accompanied by accumulation of deleterious changes (molecular and cellular damages) that result in increased risk of diseases and death [12]. The loss of maintenance of DNA integrity contributes to the onset of cellular senescence or cell death, and this genomic instability is caused by oxidative stress, genotoxic drugs and replication errors [2].

Emerging evidence has suggested that p53 family members exert powerful roles in maintaining genome stability by halting cell proliferation, and they are functionally involved in DNA damage response including cell cycle arrest and apoptosis by controlling DNA repair protein expression, which ultimately regulates cellular senescence and aging process [2, 3, 13]. In addition, these different responses of cells such as cell cycle arrest, apoptosis, or senescence have been reported to be dependent on cell type or the level of p53 when numerous p53 target genes are upregulated in many biological processes [5].

So far, a number of studies have been reported on p53-mediated senescence. Most of the studies have focused on the role of senescence program and p53 action associated with a permanent cell cycle arrest as a tumor suppressor mechanism, while some of the studies have reported the protective role of senescence and p53 action (in limiting liver fibrosis) which is related to noncancer pathology [5, 14, 15]. In addition, little is known about age-dependent change of p53 and p63 expressions in the mouse hippocampus during the normal aging. Therefore, we investigated age-related changes in p53 and p63 immunoreactivities in the hippocampus of young, adult and aged mice, as a useful model for age research [16, 17].

## Materials and methods

### Experimental animals

Male ICR mice were purchased from Orient Bio Inc. (Seongnam, South Korea) and used at postnatal month (PM) 1, PM 6 and PM 24 as young, adult and aged group, respectively, according to the definition of the life history phases of mice [18]. Mice (*n* = 7 in each group) were housed and provided free access to food and water in a conventional state under adequate temperature (about 23 °C) and humidity (about 60%) control with a 12-h light/12-h dark cycle. For animal handling and care, we complied with the guidelines of current international laws and policies (NIH Guide for the Care and Use of Laboratory Animals, The National Academies Press, 8th Ed., 2011). In addition, our experimental protocol was approved (approval no. KW-180124-2) by Institutional Animal Care and Use Committee of Kangwon National University (Chuncheon, Republic of Korea).

### Tissue processing for histology

All mice were anesthetized by a single intraperitoneal injection of 60 mg/kg pentobarbital sodium (JW Pharm. Co., Ltd., Republic of Korea). They were transcardially rinsed with 0.1 M phosphate-buffered saline (PBS, pH 7.4) (Sigma, St. Louis, MO, USA) and fixed with 4% paraformaldehyde (Sigma, St. Louis, MO, USA) in 0.1 M PBS (pH 7.4) (Sigma, St. Louis, MO, USA). Their brains were removed and postfixed with the same fixative for 5 h, and the brain tissues were cryoprotected by infiltration with 30% sucrose (Sigma, St. Louis, MO, USA) for 8 h. The brain tissues were then frozen and transversely sectioned into 30-μm thickness in a cryostat (CM1520, Leica Microsystems, Wetzlar, Germany).

### Immunohistochemistry

To examine age-related change in p53, p63 and NeuN (a marker for neurons) immunoreactivity in the mouse hippocampus, immunohistochemical stainings and their quantitative analyses were performed according to our published method [11]. We used polyclonal rabbit anti-p53 antibody (cat. no. ab131442, 1:250; Abcam, Cambridge, MA, USA), polyclonal rabbit anti-p63 antibody (cat. no. ab53039, 1:250; Abcam, Cambridge, MA, USA), and polyclonal mouse anti-NeuN (cat. no. MAB377, 1:200; Millipore, Cambridge, MA, USA) as primary antibodies. According to our method, the brain sections were reacted with each antibody at 4 °C for 8 h and followed by biotinylated goat anti-rabbit IgG or horse anti-mouse (1:200; Vector Laboratories, Burlingame, CA, USA) and streptavidin peroxidase complex (1:200; Vector Laboratories, Burlingame, CA, USA) at room temperature for 2 h. Each negative control test was carried out by using pre-immune serum (Vector Laboratories, Burlingame, CA, USA) instead of each primary antibody to establish the specificity of each immunostaining. All negative control tests showed no immunoreactivity in any cells.

According to anatomical landmarks of the mouse brain atlas, we selected seven sections with 120-μm intervals per animal and quantitatively analyzed p53, p63 and NeuN immunoreactivity, respectively. As previously described [11], we captured the digital image of each immunoreactivity in the mouse hippocampus under an AxioM1 light microscope (Carl Zeiss, Germany) equipped with a digital camera (Axiocam, Carl Zeiss, Germany) connected to a PC monitor.

P53 and p63 immunoreactive structures were captured in a 250 × 250 μm square in the hippocampus and analyzed by relative immunoreactivity with an image analyzing system (software: Optimas 6.5, CyberMetrics, Scottsdale, AZ). The immunoreactivity of p53 and p63 was evaluated by optical density (OD). OD was obtained after the transformation of the mean gray level by using a formula: OD = log (256/mean gray level). The background density was subtracted, and a ratio of OD was calibrated as % (relative OD, ROD) by using Adobe Photoshop (version 8.0). Finally, ROD was evaluated by using Image J 1.46 software (National Institutes of Health, Bethesda, MD, USA). A ratio of ROD was calibrated as %, with the young group (100%).

To evaluate neuronal distribution in the hippocampus, NeuN immunoreactive neurons were counted in a 250 × 250 μm square in the hippocampus with an image analyzing system (software: Optimas 6.5, CyberMetrics, Scottsdale, AZ). Cell counts were obtained by averaging all counts obtained from each animal.

#### Fluoro-jade B (F-J B) histofluorescence staining

To examine whether hippocampal neurons are intact or not in each group, F-J B (a fluorescent marker for cellular degeneration) histofluorescence staining was done as described previously [19]. Briefly, the brain sections were immersed in a 1% solution of sodium hydroxide (Sigma-Aldrich, MO, USA) for 5 min, transferred to a solution of 0.06% potassium permanganate (Sigma-Aldrich, MO, USA) for 20 min and incubated in a solution of 0004% F-J B (Histochem, Jefferson, AR, USA) for 45 min. Finally, the reacted sections were washed and placed on a slide warmer (approximately 50 °C) for the reaction. Images of F-J B positive cells were captured with an AxioM1 light microscope (Carl Zeiss, Göttingen, Germany) equipped with a digital camera (Axiocam, Carl Zeiss, Germany) connected to a PC monitor.

### Statistical analysis

The data shown here represent the means ± SEM. Differences of the means among the groups were statistically analyzed by analysis of variance (ANOVA) with a post hoc Bonferroni’s multiple comparison test in order to elucidate age-related differences among groups. Statistical significance was considered at *P* < 0.05.

## Results

In this study, we examined p53, p63 and NeuN immunoreactivity, and F-J B histofluorescence in the hippocampus proper, which consists of Cornu Ammonis 1–3 subfields (CA1–3), and in the dentate gyrus.

### p53 immunoreactivity

#### CA1–3

**Fig. 1:**
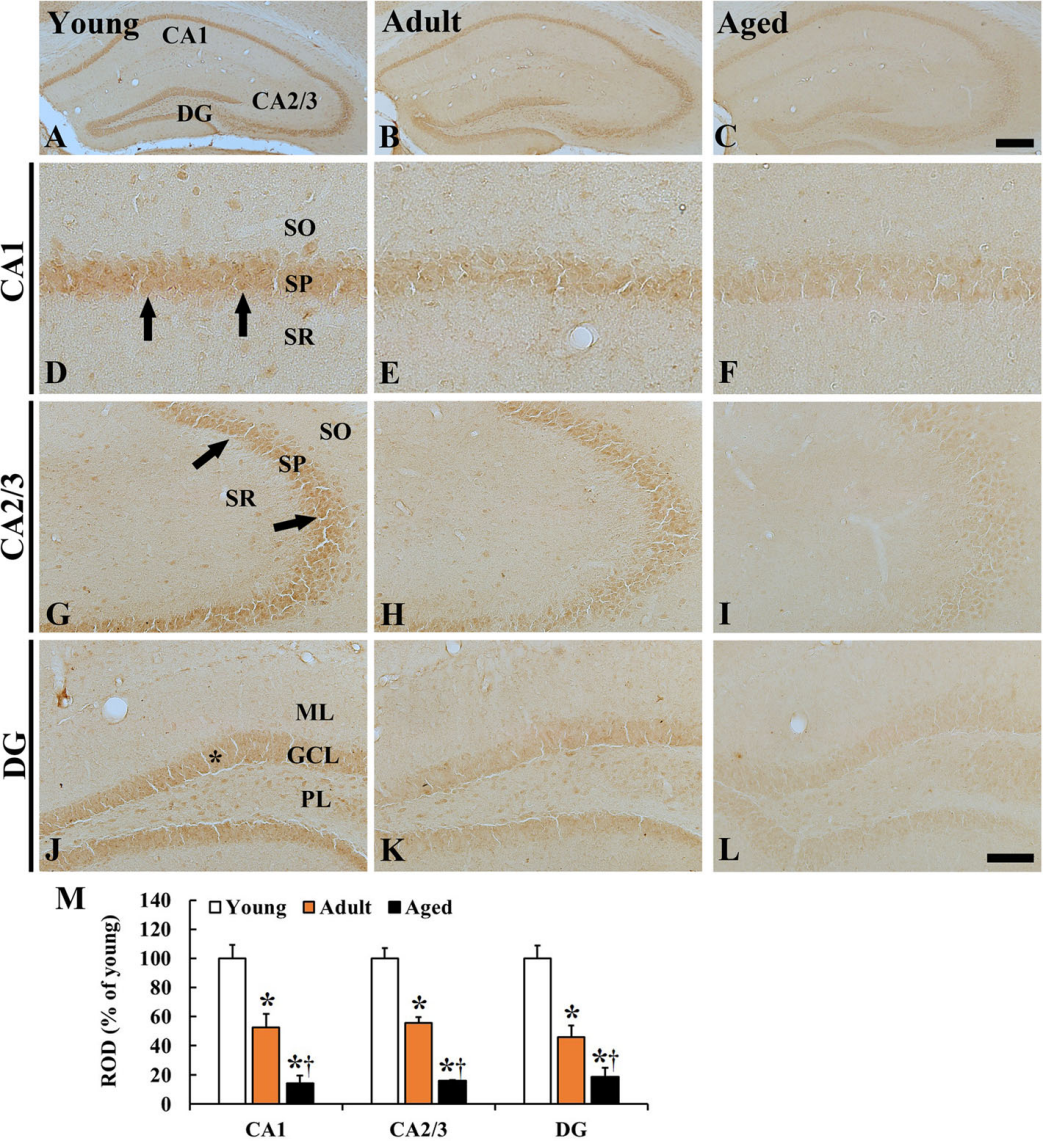
**a-l** p53 immunohistochemistry in the hippocampus of the young (**a, d, g, j**), adult (**b, e, h, k**) and aged (**c, f, i, l**) mice. p53 immunoreactivity is shown in the pyramidal cells (arrows) and granule cells (asterisk) in the young mouse hippocampus. p53 immunoreactivity in these cells is significantly and gradually decreased during aging process. GCL, granule cell layer; ML, molecular layer; PL, polymorphic layer; SO, stratum oriens; SP, stratum pyramidale; SR, stratum radiatum. Scale bar = 400 μm (A-C) and 100 μm (D-L). (M) ROD as % of p53 immunoreactive cells in the hippocampus (*n* = 7 per group; ^*^P < 0.05, significantly different from the young group, ^†^*P* < 0.05, significantly different from the adult group). The bars indicate the means ± SEM

In the young group, strong p53 immunoreactivity was detected in pyramidal cells, which consist of the stratum pyramidale, and in non-pyramidal cells, which are located in the stratum oriens and radiatum (Fig. 1 A, D, G). In the adult group, p53 immunoreactivity in the pyramidal cells was significantly decreased (52.4% in the CA1 and 55.6% in the CA2/3 of the young) (Fig. 1 B, E, H, M). In the aged group, p53 immunoreactivity in the pyramidal cells was more decreased (14.6% in the CA1 and 16.1% in the CA2/3 of the young and 27.8% in the CA1 and 18.7% in the CA2/3 of the adult) (Fig. 1 C, F, I, M).

#### Dentate gyrus

Strong p53 immunoreactivity was easily shown in granule cells, which consist of the granule cell layer, and in hilar cells, which are located in the polymorphic layer (Fig. 1 J). p53 immunoreactivity in the granule cells was significantly and gradually decreased with age. The ROD in the adult and aged was 46.1 and 18.7%, respectively, of the young (Fig. 1 K, L, M).

### p63 immunoreactivity

#### CA1–3

**Fig. 2:**
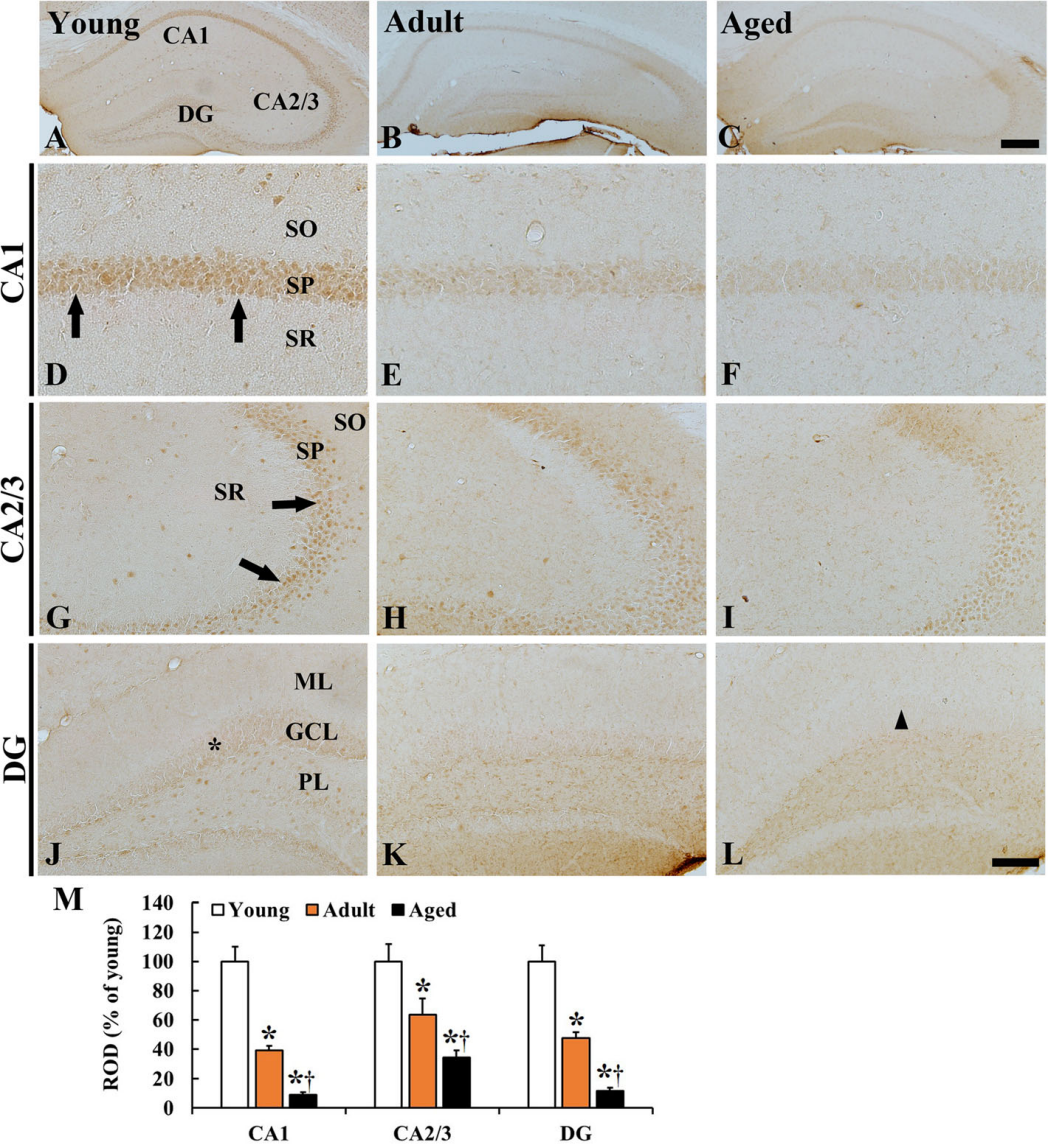
**A-L** p63 immunohistochemistry in the hippocampus of the young (**a, d, g, j**), adult (**b, e, h, k**) and aged (**c, f, i, l**) mice. p63 immunoreactivity is found in pyramidal cells (arrows) and granule cells (asterisk) in the young mouse hippocampus. p63 immunoreactivity is gradually reduced during aging, showing that p63 immunoreactivity is hardly shown in aged granule cells (arrowhead). GCL, granule cell layer; ML, molecular layer; PL, polymorphic layer; SO, stratum oriens; SP, stratum pyramidale; SR, stratum radiatum. Scale bar = 400 μm (**A-C and a-c**) and 100 μm (**D-L and d-l**). (M) Relative optical density (ROD) as % of p63 immunoreactive cells in the hippocampus (*n* = 7 per group; ^*^P < 0.05, significantly different from the young group, ^†^*P* < 0.05, significantly different from the adult group). The bars indicate the means ± SEM

In the young group, strong p63 immunoreactivity was found in pyramidal of the stratum pyramidale (Fig. 2 A, D, G). In the adult group, p63 immunoreactivity in the pyramidal cells was significantly decreased (39.1% in the CA1 and 63.5% in the CA2/3 of the young) compared to that in the young group (Fig. 1 B, E, H, M). Furthermore, p63 immunoreactivity in the pyramidal cells of the aged group was more decreased (9.1% in the CA1 and 34.1% in the CA2/3 of the young and 23.1% in the CA1 and 53.7% in the CA2/3 of the adult) (Fig. 2 C, F, I, M).

#### Dentate gyrus

In the young group, p63 immunoreactivity in the dentate gyrus was weakly shown in granule cells, and relatively strong p63 immunoreactivity was detected in hilar cells (Fig. 2 J). In the adult group, p63 immunoreactivity was significantly decreased in the granule cells, showing that the ROD of p63 immunoreactivity of the adult was 47.6% of the young (Fig. 2 K, M). In the aged group, p63 immunoreactivity in granule cells was very low, showing that the ROD of p63 immunoreactivity in the aged dentate gyrus was 11.6% of the young (Fig. 2 L, M).

### NeuN immunoreactivity

**Fig. 3:**
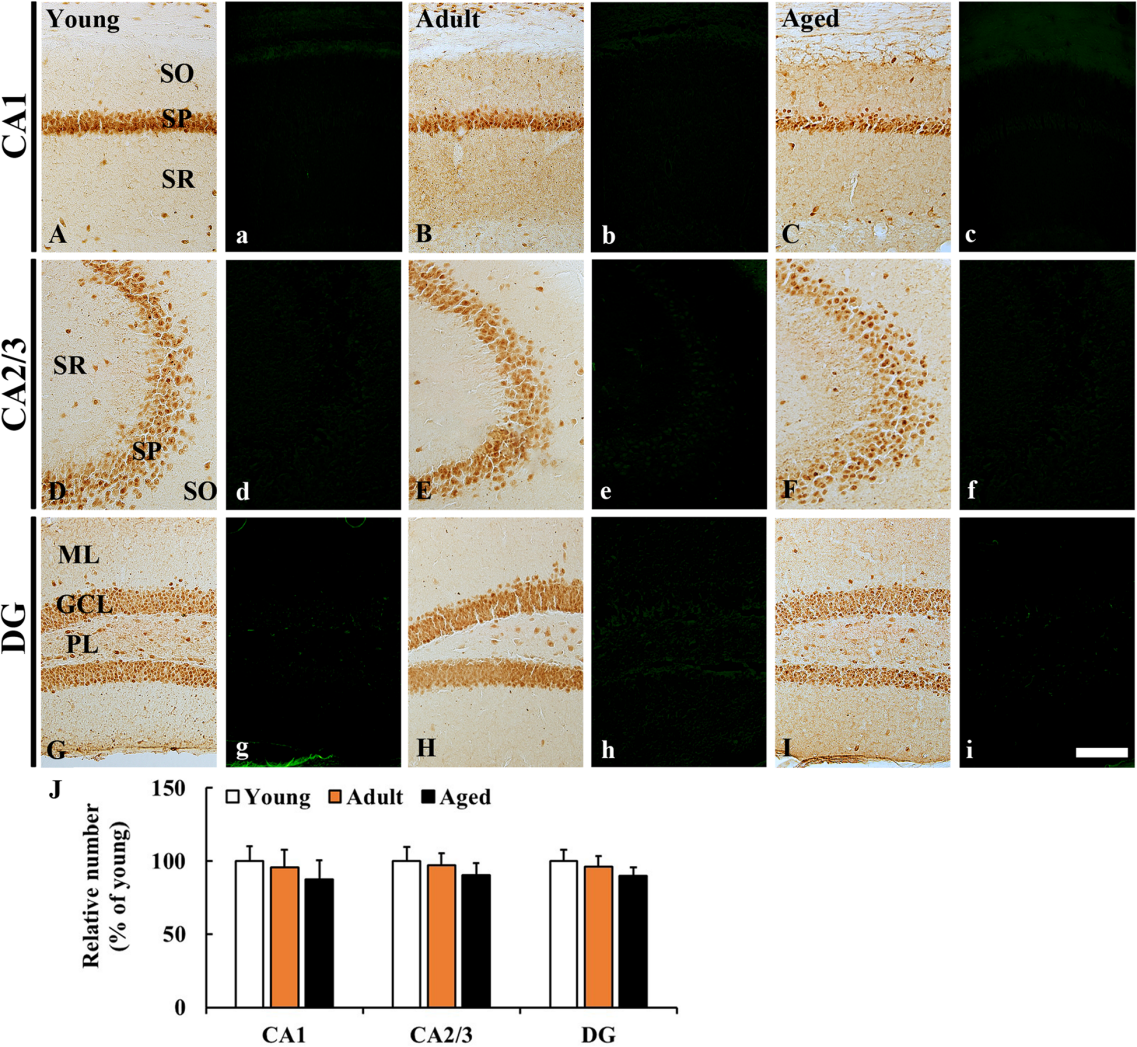
**A-I, a-i** NeuN immunohistochemistry (**A-I**) and F-J B fluorescence staining (**a-i**) in the hippocampus of the young (**A, D, G, a, d, g**), adult (**B, E, H, b, e, h**), and aged (**C, F, I, c, f, i**) mice. In all groups, NeuN immunoreactive neurons are mainly found in the stratum pyramidale (SP) and granule cell layer (GCL), and numbers of NeuN immunoreactive neurons is not significantly different among all the groups. In addition, No F-J B positive cells are detected in any groups. Scale bar = 100 μm. (**j**) The mean number of NeuN immunoreactive neurons in the hippocampus (*n* = 7 per group). The bars indicate the means ± SEM

In the young group, NeuN immunoreactive cells were mainly detected in the stratum pyramidale of the hippocampus proper (CA1–3) (Fig. 3 A, D) and in the granule cell layer and polymorphic layer of the dentate gyrus (Fig. 3 G). In the adult and aged groups, the distribution pattern of NeuN immunoreactive cells was similar to that in the young group, showing that the mean number of NeuN immunoreactive cells in the stratum pyramidale and in the granule cell layer was not significantly different from that the young group (Fig. 3 B, C, E, F, H, I, J).

### F-J B immunofluorescence

F-J B positive cells were not detected in any layers in the CA1–3 (Fig. 3 a-f) and the dentate gyrus (Fig. 3 g-i) in all groups.

## Discussion

In the present study, we found that each p53 and p63 immunoreactivity was mainly expressed in pyramidal and granule cells in the young, adult and aged mouse hippocampus. This finding is consistent with previous studies. Ding et al. (2014) have reported that specific p53 expression is shown in hippocampal pyramidal cells of young adult (6–8 weeks of age) [20] and aged (20–29 months of age) [7] rats. In addition, Napieralski et al. (1999) have reported that p53 mRNA is detected in pyramidal cells of the CA1-CA3 subregions and granule cells of the dentate gyrus of the adult rat [21]. In regard to p63 expression, we have reported that p63 immunoreactivity is shown in hippocampal pyramidal cells of young and adult gerbils [11, 22]. In addition, Hernandez-Acosta et al. (2011) have reported that, in the young mouse hippocampus, strong p63 immunoreactivity is found in pyramidal cells of the hippocampus proper (CA1–3) and hilar cells of the dentate gyrus, showing that p63 immunoreactivity in dentate granule cells is relatively weak [8]. This finding is consistent with our present study on the young mouse. Based on previous and our current studies, we insist that p53 and p63 immunoreactivity in rodent hippocampi is typically expressed in pyramidal and granule cells in the rodent hippocampus.

In our current study, p53 and p63 immunoreactivity in the mouse hippocampus was highest at young. It has been reported that p53 serves to ameliorate the disruption of neuronal development after irradiation by inhibiting neural progenitor activation in the young (10 weeks old) mouse dentate gyrus [23]. Furthermore, Hernandez-Acosta et al. (2011) have reported that p63 protein level in the young adult mouse brain is significantly higher than that at embryonic and early postnatal stage, and they suggested that p63 gene might play more important roles in neuronal maintenance in adulthood than neuronal development at early postnatal stage [8]. In addition, it has been demonstrated that p63 is essential for the development and maintenance of stratified epithelial tissues [24, 25]. Based on the results of previous and our current studies, it is postulated that strong p53 and p63 expressions in pyramidal and granule cells in the young hippocampus is related to neuronal maintenance in the hippocampus in young period.

We, in this study, found that p53 and p63 immunoreactivity in the mouse hippocampus was dramatically decreased in the aged group compared to that in the young and adult groups. This is in accordance with previous studies that show that aging is enhanced when p53 or p63 is removed by genetic modification. Namely, Moore et al. (2007) have demonstrated that truncated form of p53 protein increases wild-type p53 activity and promotes the aging process, which reduces life span [26]. Armata et al. (2007) have reported that, in p53^S18A^ mice, Serine^18^ of p53 is replaced with non-phosphorylable alanine and displays signs of accelerated aging [27]. Some researchers have reported that p63^+/−^ mice show a decreased lifespan and features of accelerated aging with decreased proliferation and enhances expressions of senescent markers such as senescence-associated-β-galactosidase, promyelocytic leukemia protein, and tumor suppressors p16^INK4a^, suggesting that p63 deficiency causes cellular senescence and organismal aging [13, 28]. Furthermore, Keyes et al. (2005) have demonstrated that p53 depletion prevents aging induced by loss of p63 in vitro*,* indicating that interaction between p53-related proteins functionally regulate the aging process [28]. Therefore, it is postulated that one of physiological p53 and p63 activities may be involved in protecting tissues from aging-associated characters and that predominantly decreased p53 and p63 immunoreactivity in the aged hippocampus may be closely related to one of features of normal aging process.

p53 and its family member p63 are important regulators in aging, and p53 can prevent or promote aging in a context-dependent manner [2, 3, 13]. Mechanisms to regulate aging and longevity by p53 include regulation of mammalian target of rapamycin (mTOR) signaling and reactive oxygen species (ROS) generation as follows. Under no or low stress condition, p53 can reduce mTOR signaling and decrease ROS by inducing antioxidant genes expression, whereas, under severe stress, active p53 increases intracellular ROS, which leads to pro-apoptotic and pro-senescent activities [3, 29]. Indeed, in our current study, we have not found age-related neuronal death/degeneration of pyramidal and granule cells in the aged mouse hippocampus with a marked reduction in p53 and p63 immunoreactivity. In addition, it has been investigated that mTOR phosphorylation is significantly decreased in the hippocampus of aged mice [30] and aged gerbils [31], and that some antioxidant, such as Cu/Zn SOD immunoreactivity is gradually increased in the hippocampal CA1 with age (from 5 to 15 months of age) in mice [32], although other antioxidant catalase immunoreactivity is decreased in the mouse hippocampus during aging [33]. On the other hand, it has been reported that significant loss of Purkinje cells in the aged rat cerebellum is closely related to upregulation of p53 in the Purkinje cells [7]. Taken together, significantly reduced p53 and p63 expression in senescent mouse hippocampus likely that normal aging is not a severe stress situation, which is closely related to absence of neuronal cell death.

## Conclusion

Our current study showed that p53 and p63 immunoreactivity in the mouse hippocampus during the normal aging process was gradually and significantly reduced in an age-dependent manner, showing no death or loss of any pyramidal cells in aged mice. These findings suggest that decrease in p53 and p63 expression may be closely related to age-associated changes in the hippocampus, and it can be clinically used as indicators of normal or abnormal aging.

## Data Availability

All data generated or analyzed during this study are included in this published article.

## Abbreviations

CA: Cornu ammonis
F-JB: Fluoro Jade B
mTOR: Mammalian target of rapamycin
OD: Optical density
ROD: Relative optical density
ROS: Reactive oxygen species
